# Novel treatment of gastric outlet obstruction secondary to incarcerated inguinal hernia: percutaneous endoscopic gastrostomy tube

**DOI:** 10.1093/jscr/rjad294

**Published:** 2023-06-19

**Authors:** Sawyer M Blair, Roger M Cournoyer, Melissa R Newcomb, Joseph W Owen

**Affiliations:** University of Kentucky College of Medicine, Lexington, KY, USA; Department of Surgery, University of Kentucky, Lexington, KY, USA; Department of Surgery, University of Kentucky, Lexington, KY, USA; Department of Radiology, University of Kentucky, Lexington, KY, USA

## Abstract

A 93-year-old man presented with gastric outlet obstruction (GOO) secondary to a massive left inguinal hernia with incarcerated antrum. He reported a desire to avoid operative intervention, and given his comorbidities, such an operation carried high risk for perioperative complications. As such, we offered percutaneous endoscopic gastrostomy (PEG) tube placement, as this would allow intermittent decompression of the stomach to reduce the risk of obstruction and strangulation. He tolerated the procedure well and was discharged after several days of observation. He continues to do well at regular outpatient appointments. Although rare, GOO secondary to an incarcerated inguinal hernia is most likely to occur in a patient such as ours: elderly, comorbid and at high risk for perioperative complications. To our knowledge, this is the first documented case to be treated with a PEG tube, which can be a desirable and effective option in this subset of patients.

## INTRODUCTION

The inguinal hernia accounts for 75% of abdominal wall hernias [1]. Omentum and small bowel are the most common intraabdominal structures to traverse the fascial defect and become vulnerable to incarceration and strangulation [2]. Less frequently, these hernias may contain appendix, bladder, ovary, oviduct, cecum or sigmoid [2]. Very rarely, part of the stomach can become incarcerated in large inguinal hernias. A recent literature review found only 21 published cases from 1942 to 2020 of inguinal hernias containing the stomach, and of those only 10 reported gastric outlet obstruction (GOO) [3]. Treatment has historically been surgical reduction of intraabdominal contents and hernia repair, with a smaller subset treated conservatively with nasogastric (NG) tube decompression [3]. This is a presentation of a case of GOO secondary to incarcerated inguinal hernia in a 93-year-old man who was successfully treated with a percutaneous endoscopic gastrostomy (PEG) tube.

## CASE PRESENTATION

**Figure 1:**
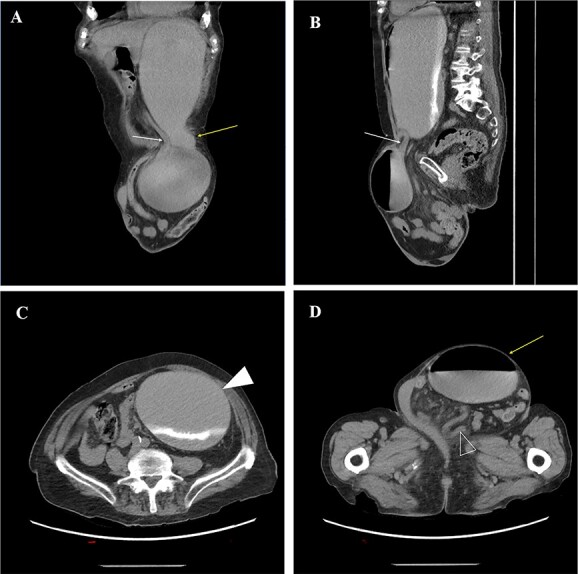
(**A**) oblique coronal reconstructions from a non-contrast CT demonstrates the gastric body (yellow arrow) entering the left inguinal hernia, and the obstructed gastric antrum (white arrow) exiting the hernia; (**B**) oblique sagittal reconstruction from a CT demonstrates the obstructed gastric antrum (white arrow); (**C**) axial non-contrast CT through the abdomen demonstrates a dilated gastric body and fundus (white arrowhead) with layering contrast fluid and ingested material; (**D**) axial non-contrast CT image through the pelvis demonstrates a large left inguinal hernia containing the obstructed gastric body (yellow arrow) and multiple loops of decompressed small bowel (open arrowhead).

A 93-year-old man presented to the emergency department complaining of intractable non-bloody, non-bilious emesis. He reported a 25-year history of a progressively enlarging left inguinoscrotal hernia associated with intermittent pain, discomfort and early satiety. Two weeks previously he had presented to the emergency department (ED) with similar symptoms but left after receiving fluids and spontaneous resolution. Physical examination revealed a massive, incarcerated left inguinal hernia which obscured the penis, focal tenderness at the hernia and no signs of peritonitis or strangulation. Computed tomography (CT) revealed a left inguinal hernia that contained loops of small bowel (Fig. 1D), and a portion of the gastric body and antrum with severe proximal dilation of the stomach (Fig. 1A), concerning for GOO. An NG tube was placed with immediate evacuation of 2L of contents. The patient noted instant relief as well as improved pain at the hernia site.

Given the patient’s history of chronic obstructive pulmonary disease and the likelihood of a laparotomy for repair of the hernia, his risk of perioperative morbidity, mortality and prolonged ventilation was high. The patient clearly described his wish to attempt the least invasive treatment option, even if risk of failure was higher. We proposed the possibility of PEG tube placement as definitive therapy in his case. A PEG tube would allow intermittent decompression of the stomach and minimize the risk of recurrent GOO. The patient agreed to the procedure.

Complete esophagogastroduodenoscopy was performed in which no pathology was noted apart from the incarcerated gastric hernia. The PEG tube was successfully placed in the gastric body under direct vision. He remained under observation for several days. He advanced to a regular diet with intermittent venting of the PEG tube performed by himself, as needed for symptoms. The patient was pleased with the result and discharged home without complications. He continues to do well at multiple follow-up visits.

## DISCUSSION

This case demonstrates two rarities: an inguinal hernia containing the stomach, and a GOO caused by mechanical impingement of the stomach in a hernia [4]. In 2021 Heylen *et al*. [3] published a systematic review of the literature containing all cases of inguinal hernias that contained the stomach. They only found 21 such cases, ranging in time from 1942 to 2020. Of these 21 cases, only 10 presented with GOO. Five of those 10 were treated conservatively with NG decompression, IV fluids and bowel rest. The others were treated with surgical repair [3]. While conservative management has shown to be a viable route for patients to recover and be discharged from the hospital, symptoms can recur [5]. A case reported in 1987 demonstrates this, with a patient having recurrent episodes of vomiting and hospital admission due to GOO secondary to an inguinal hernia [6]. In response to his recurring symptoms, this patient underwent surgical hernia repair. Indeed, our own patient in this case had recurring symptoms, having visited the ED 2 weeks prior with similar symptoms. As demonstrated, conservative treatment can resolve symptoms but does not provide a durable solution to prevent recurrence.

Surgical hernia repair is the standard definitive treatment to avoid complications from hernias such as this [7, 8]. These complications include recurrent GOO, strangulated bowel and gastric perforation in the incarcerated inguinal hernia [9, 10]. However, not every patient is willing or able to be a surgical candidate. As such, it would be beneficial to find an alternative solution to surgery or conservative therapy that decreases the risks of recurrent GOO and gastric perforation in elderly patients like ours. Our patient in this case had no signs of bowel ischemia or gastric perforation, which are surgical emergencies, and was adamant in his refusal of elective surgical repair. The solution proposed by our team was to place a PEG tube. It was hypothesized that by placing the PEG tube through the superior body of the stomach and abdominal wall, the stomach could be intermittently decompressed and avoid prolapse back into the inguinal canal. NG tube decompression is successful at relieving symptoms in a similar manner, but GOO is likely to recur when the NG is removed. With the PEG tube in place, there would be permanent intermittent access to decompress the stomach, preventing recurrent GOO for our patient while avoiding surgical repair of the hernia. The patient tolerated PEG tube placement well, and over the ensuing days tolerated a regular diet and learned to use the PEG tube for decompression. He was discharged shortly thereafter, symptom free.

To our knowledge, this is the first documented case of GOO secondary to an incarcerated inguinal hernia containing gastric antrum which was successfully treated with PEG tube placement. While exceedingly rare, GOO secondary to this etiology occurs in a predictable and consistent patient population: elderly males with comorbidities and high operative risk. Intermittent decompression with a PEG tube may offer these patients acceptable quality of life without the risks of a major operation.

## CONFLICT OF INTEREST STATEMENT

None declared.

## FUNDING

None.

## DATA AVAILABILITY

Data available within the article or its supplementary materials.
